# Effects of Pyrolysis Temperature on Product Yields and Energy Recovery from Co-Feeding of Cotton Gin Trash, Cow Manure, and Microalgae: A Simulation Study

**DOI:** 10.1371/journal.pone.0152230

**Published:** 2016-04-04

**Authors:** Muhammad Usman Hanif, Sergio C. Capareda, Hamid Iqbal, Renato Ortiz Arazo, Muhammad Anwar Baig

**Affiliations:** 1 Bio-Energy Testing and Analysis Laboratory (BETA Lab), Biological and Agricultural Engineering Department, Texas A&M University, College Station, Texas, 77843, United States of America; 2 Institute of Environmental Science and Engineering, School of Civil and Environmental Engineering, National University of Sciences and Technology, Sector H-12, Islamabad, 46000, Pakistan; 3 Institute of Engineering and Technology, Misamis Oriental State College of Agriculture and Technology, Northern Mindanao, 9004, Philippines; Center for Nanosciences and Nanotechnology, MEXICO

## Abstract

The intensive search of new and cleaner energy catches interest in recent years due to huge consumption of fossil fuels coupled with the challenge of energy and environmental sustainability. Production of renewable and environmentally benign energy from locally available raw materials is coming in the frontline. In this work, conversion of the combined biomass (cotton gin trash, cow manure, and Microalgae [*Nannochloropsis oculata*]) through batch pyrolysis has been investigated. The effect of temperature to the production of energy fuels such as bio-oil, char, and biogas have been simulated considering the yield and energy content as responses. Result of the investigation generally revealed that the proportions of the different biomass did not significantly affect the product yield and energy recovery. Significant effect of temperature is evident in the simulation result of energy recovery whereby maximum conversion was achieved at 400°C for char (91 wt%), 600°C for syngas (22 wt%), and 551°C for bio-oil (48 wt%). Overall energy conversion efficiency of 75.5% was obtained at 589°C in which 15.6 MJ/kg of mixed biomass will be elevated to pyrolysis products.

## 1. Introduction

After several decades of industrialization powered by the consumption of conventional fuels, the world is now facing tremendous crisis on energy resource. The increasing demand for primary energy coupled with the diminishing reserve of fossil fuels pose an alarming global situation of energy predicament [1]. Current directions are now geared towards energy production from alternative sources that are sustainable, affordable, and environmentally benign. One of the fields under thorough investigation is the use of biomass for energy production. Biomass from agricultural and industrial wastes such as rice husk [2], cotton gin trash [3–5], rice bran [6], [7], bagasse [8], [9], and de-oiled seed cakes [10], [11], have been found promising substitutes to crude oil in various applications. However, this kind of biomass can still be found discarded in the environment which, aside from opportunity cost wasted, possibly poses detrimental effect to the environment due to associated pollution generated.

Pyrolysis is one of the emerging technologies that utilize different kinds of biomass for biofuel production. With pyrolysis, organic wastes can be substantially reduced with an increase economic profit from the use of whole product chain. In the process, organic by-products of agriculture and industry are converted into more valuable biofuel products such as solid char, liquid bio-oil, and syngas [12]. These pyrolysis products have been found to have various applications [13]. The liquid bio-oil product for example is a potential feedstock to produce hydrocarbon/bio gasoline [14] that could potentially substitute the petroleum-based gasoline for transportation and stationary engines.

Though pyrolysis of some biomass from agriculture and industries has been explored [15], [16]; very limited work investigated the co-feeding of the different locally available biomass. Previous investigations [17] focused more in converting single biomass to biofuels. This conventional approach of one-biomass-at-a-time is generally less applicable because of the unsustainable source of specific biomass that can be used in the large-scale application. This limitation, however, can be addressed by co-feeding different kinds of biomass. In this way, a variety of locally available biomass in one area can be utilized.

This work investigated the applicability of co-feeding three kinds of biomass such as cow manure, cotton gin trash, and Microalgae (*Nannochloropsis oculata*) to maximize the production of pyrolysis products under different temperatures. Particularly, this work investigated the effects of temperature in the yields of char, bio-oil, and syngas. It also investigated how the temperature affected the energy contents and of the pyrolysis products. Also, this work determines the best condition for optimum energy conversion efficiency.

In order to most accurately know the effects of temperature, regression analysis was used to model its effects to pyrolysis products yields, energy recovery, and energy conversion efficiency. This analysis method is likewise used in many pyrolysis studies to develop models in predicting product yields, optimize the profit from bio-oil, and char production and design for an industrial process [18–20].

## 2. Materials and Methods

### 2.1. Biomass preparation

Three different biomass used in the co-pyrolysis experiment were collected: cotton gin trash (CGT) from Varisco Cotton Gin near College Station, Brazos County, Texas; cow dung from dairy farm of Texas A&M Animal Science Research and Extension Science Complex (ASTREC) in the City of Bryan, Texas; and dewatered Microalgae (*Nannochloropsis oculata*) from the algae pond of Texas Agri-Life Research in Pecos, Texas. To attain the biomass from these three locations, permission was granted to Bio-Energy Testing and Analysis Laboratory (BETA Lab), Texas A&M from the sub units of Texas A&M University, and from Varisco Cotton Gin. All biomass were dried separately in an oven at 105°C [21] till the time when moisture content was reduced to a level of less than 10% by weight. Cotton gin trash, cow manure, and Microalgae took one, three, and five days respectively for drying. The dried biomasses were then grounded using Wiley Laboratory Mill (Model No. 4, Arthur Thomas Company, PA). The 2 mm particle size of all biomass used in this work were obtained by sieving following ASTM E-11 (Fisher Scientific Company, USA).

### 2.2. Co-pyrolysis of biomass

In each batch experiment, 300 g of mix samples (co-biomass) were prepared following the proportions in the treatment of this study (Table 1). Microalgae was kept constant in this work because of its high heating value that could have strong potential of producing products possessing desirable heating value. The proportion of cow manure and cotton gin trash was designed considering their availability.

**Table 1:** Proportion of biomass in each treatment.

**Biomass**	**HHV (MJ/kg)**	**Treatments (%)**				
		1	2	3	4	5
Cotton gin trash	17.9	30	32.5	35.0	37.5	40
Cow manure	19.8	20	17.5	15	12.5	10
Micro-algae	23.3	50	50	50	50	50

Co-pyrolysis of biomass mixture was carried out in a batch pressure reactor (Series 4580 HP/HT, Parr Instrument Company, Moline, IL). Diagram of this set-up is already reported elsewhere [15]. Experiments were carried out at different temperatures: 400, 450, 500, 550, and 600°C. The reactor was heated at approximately 5°C/min till the desired temperature was achieved. The reaction was carried out for 30 min at the desired temperature.

The bio-oil, char, and biogas yields were expressed as weight percentage of the total amount of biomass sample used in each run. Syngas yield was computed based on the gas volume produced which was converted into mass based on the gas composition of the sample. The volume of gas produced was measured using a gas meter (METRIS 250, Itron, Owenton, KY).

High heating values of biomass (cow manure, cotton gin trash, microalgae), bio-oil (aqueous and organic phases) and char were determined using Parr Isoperibol Bomb Calorimeter (Model 6200, Parr Instrument Company, Moline, IL) following ASTM standard D5865. The heating value of gas was determined by molar concentration of syngas components. The volumetric composition of gas was determined by gas chromatography and the heating values were measured at actual temperature and pressure conditions and corrected to normalized temperature and pressure using ideal gas law [22].

### 2.3. Simulation

Pyrolysis product yields and energy recovery were modeled as a function of operating temperature. Statistical analyses were performed to verify how well the fit of the statistical model. Models were then used in simulation to forecast the yields and energy contents of pyrolysis products at different temperatures from the co-biomass.

For energy balance, the energy input to the system was computed based on the biomass gross calorific value. Energy output is the total energy from all pyrolysis products based on HHV. The amount of energy recovery in the product and the overall energy conversion efficiency were calculated in terms of percentage using Eqs (1) and (2), respectively. Optimum conditions for percentage energy recovery and energy conversion efficiency were also evaluated by OptQuest tool.

$$\text{Percent energy recovery}\;=\;\frac{\text{Wp}\;*\;\text{HVp}}{\text{Wb}*\text{HVb}}\left(100\right)$$ (1)

$$\text{Energy conversion efficiency}\;=\;\frac{\text{energy output}}{\text{energy input}}\left(100\right)$$ (2)

The *W*_*b*_ is the weight of input biomass (kg), *W*_*p*_ is the weight of pyrolysis product (kg), *HV*_*b*_ is the gross heating value of biomass (MJ/kg), *HV*_*p*_ is the gross heating value of pyrolysis product (MJ/kg), energy input is ∑(*W*_*b*_ * *HV*_*b*_) for all biomass mixture components, and energy output is ∑(*W* * *HV*) for all pyrolysis products.

Analysis of variance (ANOVA) was employed to evaluate the significant difference between treatments. Linear and binomial regression models were used to predict the energy recovery from different co-biomass at variable temperatures. Microsoft excel interfaced with crystal ball was used for modeling and simulation.

## 3. Results and Discussion

**Fig 1:**
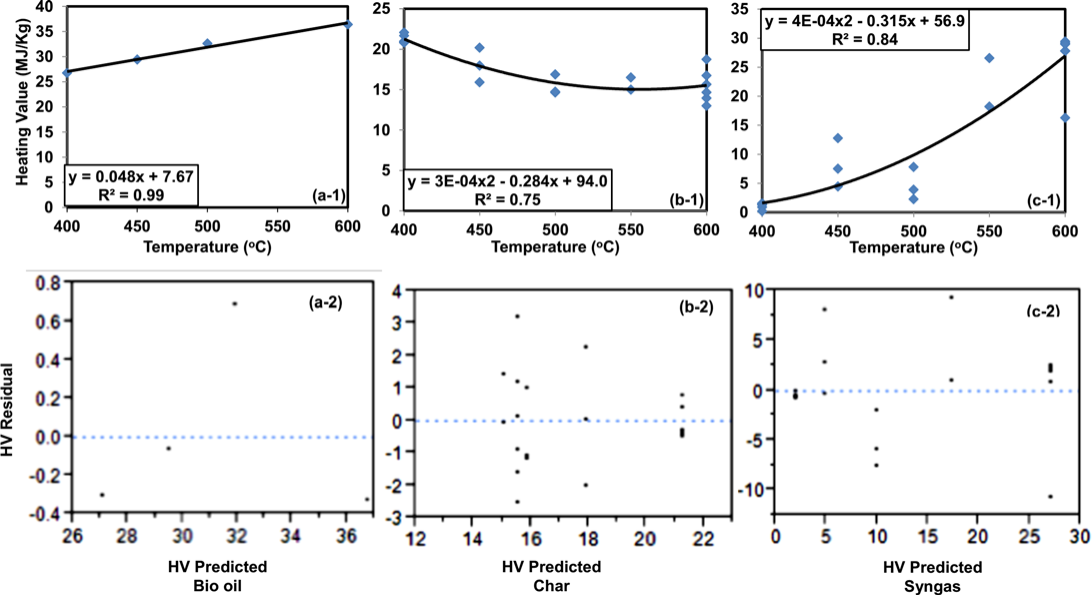
Energy content models of pyrolysis product s [a-1 Bio oil, b-1 Char, and c-1 Gas] and residual plots of models generated [a-2 Bio oil, b-2 Char, and c-2 Gas].

**Fig 2:**
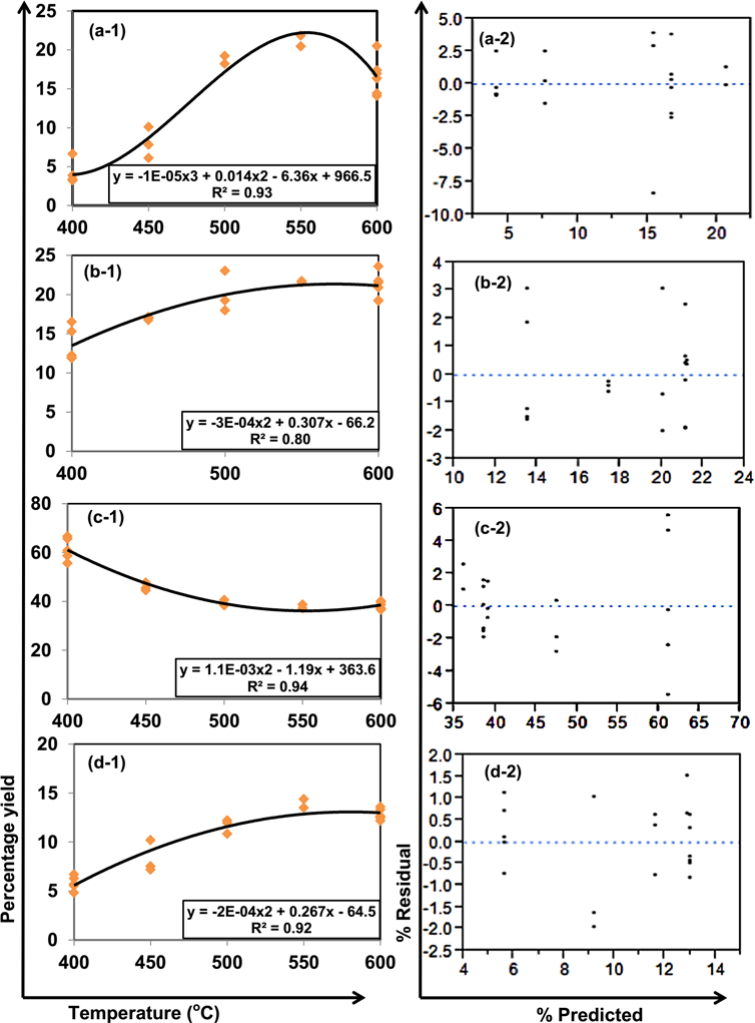
Pyrolysis yields models [a-1 Bio oil, b-1 Aqueous, c-1 Char, and d-1 Gas] and residual plots of models generated [a-2 Bio oil, b-2 Aqueous, c-2 Char, and d-2 Gas].

ANOVA result at 95% confidence level indicated that pyrolysis yield and energy contents are affected only by temperature irrespective of the mixing ratio of co-biomass. Hence, only the effect of temperature to pyrolysis energy content and yields are highlighted in this article (Fig 1 and Fig 2).

### 3.1. Effects of temperature to pyrolysis energy content

Regression models of the products’ high heating values (MJ/kg) with respective coefficient of determination (R^2^) and residual plots were obtained (Fig 1). The result showed high R^2^ values indicating best fit regression lines with the data, contrary to residual plots which cannot reasonably be used as predictive models for lack of pattern. Generally increasing trends of the heating values of bio-oil (organic + aqueous parts) and syngas products were observed with an increase in temperature.

The increase of heating value of the bio-oil at temperature up to 600°C can largely be attributed to optimum extraction of the combustible elements from the biomass, particularly carbon and hydrogen during pyrolysis course, which are favorably elevated to the bio-oil product. Similarly, the elevating heating value of syngas may be due to secondary tar reactions of the volatiles, such as thermal cracking [23], that favors the increase of gas yield [24]. Because of the increase in gas yield at high temperature, the char yield subsequently decreased as the operating temperature increases. This is an indication that, at higher temperature such as 600 °C, the energy contents of the biomass were elevated to liquid and gas products. Similar trends for the pyrolysis yields of different biomass were also observed by many researchers [15], [16], [25], [26]. The trends for heating values of pyrolysis products were also the same as were observed from the yields.

### 3.2. Effects of temperature to pyrolysis yields

Like heating value, the product yields were best illustrated using the R^2^ due to poor residual plot illustrations which could not become a tool in predicting the pyrolysis yields in the co-pyrolysis of cotton gin trash, cow manure and microalgae.

The organic and aqueous products peaks their yields at 550°C (Fig 2A-1 and 2B-1). At this temperature, maximum liquid yield up to 70 wt% of the organic solid has been reported [27]. Beyond 500°C, yield of liquid decreases with temperature because of the secondary tar reactions of the volatiles, such as thermal cracking [23], that favors the increase of gas yield [24] (Fig 2D-1). On the other hand, low pyrolysis temperature (300°C) converts up to 90 wt% of the original organic solids to char [27] (Fig 2C-1). From low to moderate temperature, the bio-oil yield rises resulting to a decrease in char yield.

### 3.3. Simulation of yields and energy content distribution

Because the amount of biomass mixtures did not have any effect on the pyrolysis yields and energy content, the quantity of biomass mixtures was defined as stochastic variables. The pyrolysis temperature was set as a decision variable in order to study the effect of changing temperature on the prediction results. This simulation can be used to predict the pyrolysis yields and heating values of products at a specified temperature and sample amount. The calculation was based on the models obtained from the previous section. An example of simulation results for pyrolysis product yield (kg) and energy recovery (MJ) is shown in Table 2.

**Table 2:** Simulation results for 1.2 kg of co-biomass at 600°C.

Description	Forecast yields (kg)				Forecasted energy contents (MJ)			
	Organic	Aqueous	Char	Syngas	Organic	Char	Syngas	Total
Trials	1000	1000	1000	1000	1000	1000	1000	1000
Base case	0.18	0.23	0.42	0.14	6.62	6.46	3.78	16.86
Mean	0.2	0.25	0.46	0.16	7.34	7.18	4.2	18.72
Median	0.2	0.25	0.46	0.16	7.34	7.17	4.2	18.7
Mode	—	—	—	—	—	—	—	—
Standard deviation	0.01	0.01	0.03	0.01	0.42	0.41	0.24	1.06
Variance	0	0	0	0	0.17	0.16	0.06	1.12
Skewness	-0.01	-0.01	-0.01	-0.01	-0.01	-0.01	-0.01	-0.01
Kurtosis	2.77	2.77	2.77	2.77	2.77	2.77	2.77	2.77
Coeff. of variation	0.06	0.06	0.06	0.06	0.06	0.06	0.06	0.06
Minimum	0.16	0.21	0.38	0.13	6.03	5.9	3.45	15.38
Maximum	0.23	0.29	0.53	0.18	8.46	8.26	4.84	21.56
Range width	0.07	0.08	0.15	0.05	2.42	2.37	1.39	6.18
Mean std error	0	0	0	0	0.01	0.01	0.01	0.03

Based on the simulation results of 1.2 kg co-biomass at 600°C, the average product yield remained 0.2 kg of organic, 0.25 kg of aqueous parts, 0.46 kg of char, and 0.16 kg of syngas. This accounts for 16.7% of organic, 20.8% of aqueous parts, 38.3% of char, and 13.3% of syngas. The product yield percentages obtained from the simulation were similar to the results in experimental runs. Note that the losses were accounted for the percentage calculations.

Further, result showed that 18.72 MJ of the biomass energy was elevated and distributed to the pyrolysis products. The high heating values of the bio-oil (7.34 MJ/kg) and the char (7.18 MJ/kg), which were forecasted to have high percentage yields, imply that the main energy from the biomass were recovered and concentrated to these two products.

### 3.4. Energy recovery and conversion efficiency

**Fig 3:**
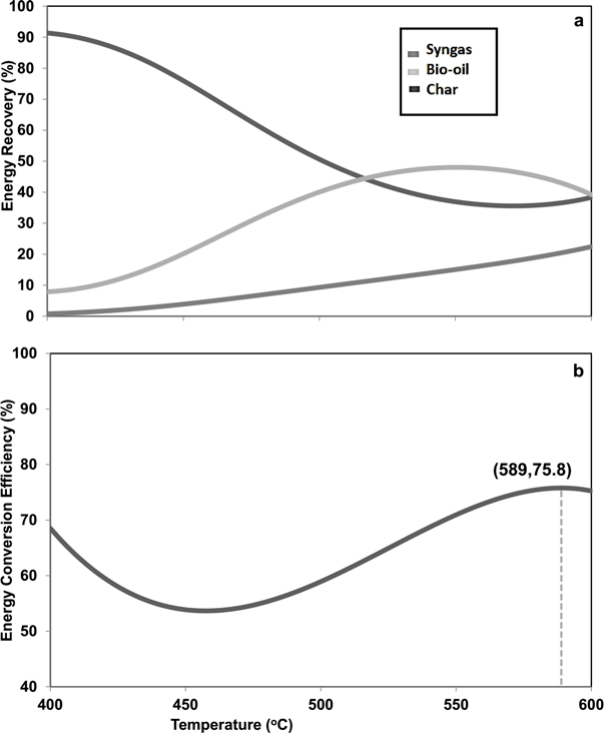
Energy recovery [a] and energy conversion efficiency [b] at different temperatures.

The decision table tool in Crystal Ball was performed by setting the operating temperature from 400°C to 600°C. The predicted energy recovery for each product and the energy conversion efficiency were obtained and plotted (Fig 3). Highest energy recovery of char was observed at the lowest temperature, which then decreased significantly when the temperature increased. On the other hand, the energy recovery for syngas remarkably increased from 400°C to 600°C. In addition, it was also found that the energy recovery for bio-oil increased from 400°C to approximately 550°C. Then it tended to decrease from 550°C to 600°C. The mixing of co-biomass altered the heating values of product by changing their respective compositions.

The energy conversion efficiencies (Fig 3B) at different operating temperatures were simulated based on the energy input of co-biomass and energy output of pyrolysis products (Fig 3A). The energy conversion efficiency decreased from 400°C to around 450°C. This can be explained by very small amounts and heating values of bio-oil observed at low temperatures as represented in Figs 1 and 2. Moreover, the reduction in char yield and its heating value could lead to the decrease in conversion efficiency because char was a major product generated at lower temperatures. However, the energy conversion efficiency tended to rise from 450°C to nearly 600°C. Even though the char yield decreased as the temperature increases, more bio-oil and syngas were produced at higher temperatures. Additionally, the heating value of bio-oil was considerably greater compared to char at high temperatures.

### 3.5. Simulation of energy recovery

**Fig 4:**
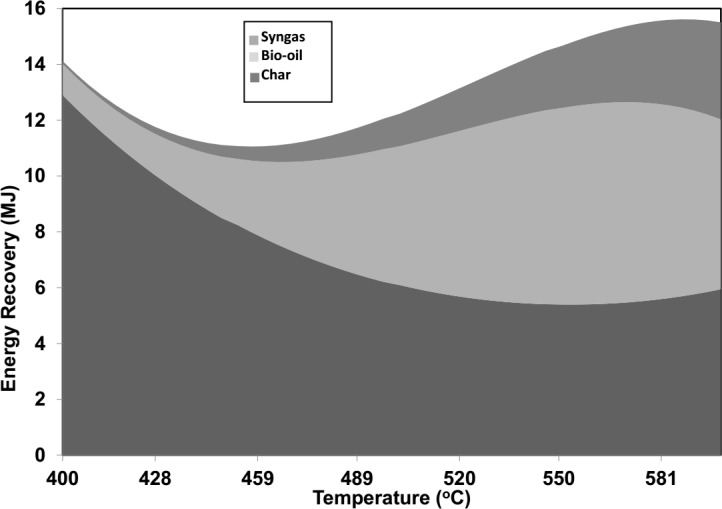
Energy production (MJ) at different temperatures.

Fig 4 shows simulation results of the energy generated from each pyrolysis product at different operation temperatures by using one kg of input biomass mixtures each run. According to the graph, the total energy produced ranged 11–16 MJ/kg approximately for the operating temperatures between 400–600°C. It can be seen that char appeared to be the main energy source especially at lower temperatures. The energy recovery from bio-oil became a major portion at temperatures higher than 500°C. A small amount of energy was produced from syngas in comparison to the energy contents obtained from char or bio-oil.

The optimum condition for energy recovery of each pyrolysis product and the energy conversion efficiency was determined. Based on the simulation results, the maximum energy recovery for pyrolysis products was achieved at 400°C for char (91%), 600°C for syngas (22%), and 551°C for bio-oil (48%). The overall energy conversion efficiency of 75.5% was obtained at 589°C. Table 3 shows results from the forecasting simulation at optimum condition (589°C). Average overall energy conversion efficiency reached 75.6%, which can be identified as 27.6% from char, 32.5% from bio-oil, and 15.5% from syngas. The decomposition of biomass through pyrolysis is a complex series of reactions. The reactions are further complexed when co-biomass is used. The reactions change with temperature, pre-treatment, biomass properties, and available moisture [28]. In our case, hydrocarbons remained in-tacked with solids other than releasing as gases. This is the reason heating values of gas at high temperature is low. The volatilization of hydrocarbons at high temperatures consequently increased the heating values of bio-oil and char.

**Table 3:** Forecasting values of energy recovery and energy conversion efficiency at optimum condition.

**Description**	**Forecast energy contents (%)**			**Forecast energy conversion efficiency**
	Organic	Char	Syngas	
Trials	1000	1000	1000	1000
Base Case	33.8	28.7	16.1	78.6
Mean	32.5	27.6	15.5	75.6
Median	32.4	27.6	15.4	75.4
Mode	—	—	—	—
Standard deviation	2	1.7	0.9	46
Variance	3.9	2.8	0.9	21
Skewness	0.3	0.3	0.3	0.3
Kurtosis	3.1	3.1	3.1	3.1
Coefficient of variation	0.1	0.1	0.1	0.1
Minimum	27.7	23.6	13.2	64.5
Maximum	39.7	33.7	18.9	92.3
Range width	12	10.2	5.7	27.8
Mean std error	0.1	0.1	0.0	0.1

## 4. Conclusions

The applicability of co-feeding cow manure, cotton gin trash, and Microalgae (*Nannochloropsis oculata*) has been thoroughly investigated in this work. Results showed that the chosen proportions of each biomass, with microalgae dominating the mixture (50 wt%), did not significantly affect the chosen responses such as yields, energy content, and energy recovery efficiency during batch pyrolysis. Temperature, on the other hand, showed significant effects of these responses. The production of char can be optimized at lower temperature (400°C) with yield reaching up to 91 wt%. The bio-oil and syngas production peaked at 551°C and 600°C with yields reaching 48 wt% and 22 wt%, respectively. The same trend was observed in the energy content and energy efficiency of the process. Char’s energy content and conversion efficiency were decreasing with increase in temperature. In contrary, the trend of the syngas energy content and conversion efficiency is increasing with temperature. Bio-oil trend showed increasing trend up to 550°C and tended to decrease beyond. Simulation study revealed that the highest yield (summation of solid char, liquid bio-oil, and syngas products) can be achieved at 589°C in which 75.5% of the energy of the biomass (20.4 MJ/Kg) was elevated to the pyrolysis products (5.8 MJ/kg from char, 6.7 MJ/kg from bio-oil, and 3.1 MJ/kg from syngas).

## Supporting Information

S1 Sheet The basic data set of product yield with High heating value. (XLSX)
