# Correction: Effects of Pyrolysis Temperature on Product Yields and Energy Recovery from Co-Feeding of Cotton Gin Trash, Cow Manure, and Microalgae: A Simulation Study

**DOI:** 10.1371/journal.pone.0156565

**Published:** 2016-07-05

**Authors:** Muhammad Usman Hanif, Sergio C. Capareda, Jinjuta Kongkasawan, Hamid Iqbal, Renato Ortiz Arazo, Muhammad Anwar Baig

Dr. Jinjuta Kongkasawan should be included in the author byline. She should be listed as the third author, and her affiliation is 1: Bio-Energy Testing and Analysis Laboratory (BETA Lab), Biological and Agricultural Engineering Department, Texas A&M University, College Station, Texas, 77843, United States of America. The contributions of this author are as follows: Conceived and designed the experiments, performed the experiments, analyzed the data, contributed reagents/materials/analysis tools.

The correct citation is: Hanif MU, Capareda SC, Kongkasawan J, Iqbal H, Arazo RO, Baig MA (2016) Effects of Pyrolysis Temperature on Product Yields and Energy Recovery from Co-Feeding of Cotton Gin Trash, Cow Manure, and Microalgae: A Simulation Study. PLoS ONE 11(4): e0152230. doi:10.1371/journal.pone.0152230
